# Research on reinforcement learning-based safe decision-making methodology for multiple unmanned aerial vehicles

**DOI:** 10.3389/fnbot.2022.1105480

**Published:** 2023-01-10

**Authors:** Longfei Yue, Rennong Yang, Ying Zhang, Jialiang Zuo

**Affiliations:** Air Traffic Control and Navigation College, Air Force Engineering University, Xi'an, China

**Keywords:** multi-UAV, constrained Markov decision process, SAC-Lagrangian, transfer learning, reinforcement learning

## Abstract

A system with multiple cooperating unmanned aerial vehicles (multi-UAVs) can use its advantages to accomplish complicated tasks. Recent developments in deep reinforcement learning (DRL) offer good prospects for decision-making for multi-UAV systems. However, the safety and training efficiencies of DRL still need to be improved before practical use. This study presents a transfer-safe soft actor-critic (TSSAC) for multi-UAV decision-making. Decision-making by each UAV is modeled with a constrained Markov decision process (CMDP), in which safety is constrained to maximize the return. The soft actor-critic-Lagrangian (SAC-Lagrangian) algorithm is combined with a modified Lagrangian multiplier in the CMDP model. Moreover, parameter-based transfer learning is used to enable cooperative and efficient training of the tasks to the multi-UAVs. Simulation experiments indicate that the proposed method can improve the safety and training efficiencies and allow the UAVs to adapt to a dynamic scenario.

## 1. Introduction

In recent years, unmanned aerial vehicles (UAVs) have achieved remarkable success in civilian fields, such as disaster management (Erdelj and Natalizio, 2016), agriculture protection (Faical et al., 2016), power line detection (Li et al., 2008), wireless relay networks (Ouyang et al., 2014; Shi et al., 2020), and intelligence, surveillance, and reconnaissance (Liu et al., 2019; Zhao et al., 2020). However, it is challenging for a single UAV to accomplish tasks in highly complex, confrontational, and uncertain environments due to the inherent shortcomings of small platforms, light payloads, and limited functionality. A system with multiple cooperating UAVs (multi-UAV) is more flexible and resilient to uncertainties and can accomplish complex, dull, dirty, and dangerous tasks. The Unmanned Systems Integrated Roadmap released by the US Department of Defense states that multi-UAV systems are an essential trend for future UAV development (Wineefeld and Kendal, 2011). Therefore, multi-UAV cooperative decision-making has become a hot research topic.

Various scholars have extensively studied cooperative decision-making for the multi-UAV systems. There are three major approaches: rule-based (Gaertner, 2013; Ernest et al., 2016), search-based (Ramirez et al., 2016; Zhen et al., 2018), and learning-based approaches (Zhang et al., 2019; Sun et al., 2020). A rule-based approach relies on expert knowledge to make decisions by abstracting a problem-specific logic tree with an IF-THEN structure. Gaertner (2013) studied air-combat tactics for a UAV swarm, constructed a simple rule set, and used agent-based modeling in a simulation analysis, which identified some new swarm tactics. Ernest et al. (2016) developed a two-vs.-four air-combat decision model based on genetic fuzzy trees called ALPHA. It defeated a professional pilot in simulated air combat. However, expert knowledge is incomplete and hard-coded rules are limited such that this approach is not flexible enough to cope with complex scenarios beyond expert knowledge.

Search-based methods use some parallel search and iteration mechanisms to find suboptimal solutions to an explicit objective function. Ramirez et al. (2016) used a multiobjective genetic algorithm to find a Pareto front solution to a multi-UAV mission-planning problem. Zhen et al. (2018) proposed an intelligent self-organizing ant colony optimization algorithm for multi-UAV cooperative search-and-attack mission planning. It can find optimal waypoints. However, this approach requires explicit objective functions and performs an online search. Therefore, the real-time performance of search-based methods is poor. As the problem becomes more complex, the optimal solution also becomes harder to find.

Learning-based methods apply reinforcement learning (RL), a data-driven approach in which an agent learns the optimal policy by trial and error. A neural network is used to map states and actions. Zhang et al. (2019) proposed a three-vs.-three air-combat method based on multi-agent RL. They trained multiple agents in a swarm air-combat environment and used scenario transfer training and self-play to improve the learning efficiency of the multi-agent RL. Sun et al. (2020) constructed an air-combat model based on a multi-agent hierarchical policy gradient algorithm, which outperformed other air-combat methods in terms of the agents' offensive and defensive abilities. Learning-based methods use offline training and online testing frameworks, which can achieve near real-time performance. Neural networks are used for high-dimensional-state feature extraction and hence learning-based methods are more suitable for complex decision-making scenarios.

However, there are three issues with the latest learning-based methods. First, they suffer from safety issues. The standard RL algorithm maximizes only the sum of rewards during exploration without considering unsafe behaviors, which is unacceptable in many safety-critical domains. For example, a UAV that interacts with the environment should never perform an unsafe action while exploring, such as flying into a hazardous area. For a multi-UAV formation, losing one UAV may lead to mission failure. Therefore, it is essential to improve the safety of multi-UAV systems. Second, training inefficiency is also a critical problem, one that is limiting the practical application of RL. Third, current research on multi-UAV cooperative decision-making focuses on air-to-air missions. In contrast, air-to-surface missions have not been widely studied. The above three points are the primary motivation of this study.

In this article, we studied a multi-UAV decision-making problem for an air-to-surface mission, in which multiple heterogeneous UAVs are expected to accomplish complex tasks efficiently and safely through cooperation. Each UAV is modeled as a constrained Markov decision process (CMDP; Altman, 1999). An improved soft actor-critic-Lagrangian (SAC-Lagrangian) algorithm (Ha et al., 2020) is used to solve the optimal policy. Moreover, considering the similarity and increasing difficulty of the cooperative tasks performed by the multiple UAVs, transfer learning (TL; Glatt et al., 2016) is used to improve the training efficiency. Our experimental results demonstrate the effectiveness, generalization, and safety of the proposed method, which we call the transfer-safe soft actor-critic (TSSAC) method. The main contributions of this study are as follows:

1. An end-to-end safe RL algorithm is proposed for the UAV decision-making problem. It maximizes task performance while guaranteeing safety.
2. To make the algorithm tunable for more complex environments, damping is added to address the oscillations of the standard Lagrange multiplier in the SAC-Lagrangian algorithm.
3. A parameter-based TL method is applied to improve the training efficiency of the multiple UAV agents when performing tasks. An agent can learn difficult tasks based on simpler tasks.

The remainder of this study is structured as follows. We introduced the theoretical background of RL and TL. Next, the CMDP model built for the UAV decision-making problem is presented, followed by the proposed TSSAC method. Then, the simulation experiments conducted to test the safety, training efficiency, and generalization of the proposed method are detailed, followed by the final section that concludes this study and discusses future work.

## 2. Background

### 2.1. Reinforcement learning

A branch of machine learning, RL uses the experience gained by interacting with the environment *via* reward feedback to improve a system's ability to make decisions (Littman, 2015). RL is characterized as an interaction between an agent and an environment, which can be modeled as a Markov decision process. The agent's goal is to maximize the expected return in the entire decision-making process, i.e., to find the optimal policy. Therefore, RL is a decision optimization method.

### 2.2. Maximum entropy RL

Maximum entropy RL maximizes the expected return while maximizing the entropy of the policy. It incorporates the entropy of the stochastic policy into the reward to encourage exploration.

Therefore, the policy loss can be described as follows:


(1) $$\begin{array}{l} J_{\pi }\left(\theta \right)=\overset{T}{\underset{t=1}{\sum }}\underset{\left(s_{t},a_{t}\right)\sim \rho_{\pi_{\theta }}}{E}\left[\gamma^{t}r\left(s_{t},a_{t}\right)+\alpha H\left(\pi_{\theta }\left(▪|s_{t}\right)\right)\right], \end{array}$$

where *J* is the loss or objective function, θ represents the parameters of the policy, $H\left(\pi_{\theta }\left(▪|s_{t}\right)\right)=-\log\left(\pi_{\theta }\left(a_{t}|s_{t}\right)\right)$ is the entropy of the policy, and the temperature parameter α controls how important the entropy term is. Entropy maximization leads to policies that can explore more and capture multiple modes of near-optimal policies. For example, if multiple actions seem to be equally good, the policy should assign to each action an equal probability of being chosen.

### 2.3. CMDP

Like a Markov decision process (Baxter, 1995), CMDP is formally defined as a tuple 〈*S, A, p, r, c, C*, γ〉, with a continuous and bounded state space *S*, a continuous and bounded action space *A*, a state transition function *p*:*S* × *A* × *S* → *R* that represents the transition probabilities from a state by taking an action to the next state, a reward function *r*:*S* × *A* → [*r*_min_, *r*_max_] that indicates the instant reward after taking action *a* ∈ *A* in the state *s* ∈ *S*, a cost function *c*:*S* × *A* → [*c*_min_, *c*_max_] for evaluating the safety constraint, a given cost threshold *C*, and a discount factor γ ∈ [0, 1). The goal of the agent is to learn a policy that maximizes the expected return for each episode such that the costs of a constraint violation remain below the given threshold *C* (Ray et al., 2019):


(2) $$\begin{array}{l} \{\begin{array}{l} \underset{\pi }{\max}\;\underset{\left(s_{t},a_{t}\right)\sim \rho_{\pi }}{\text{E}}\left[\underset{t}{\sum }\gamma^{t}r\left(s_{t},a_{t}\right)\right],\\ \text{s.t. }\underset{\left(s_{t},a_{t}\right)\sim \rho_{\pi }}{\text{E}}\left[\underset{t}{\sum }\gamma^{t}c\left(s_{t},a_{t}\right)\right]\leq C, \end{array} \end{array}$$

where ρ_π_ is the trajectory distribution, and the expectation of long-term costs generated by the feasible policies is less than or equal to *C* (Yang et al., 2021).

The constrained optimization problem of CMDP in the maximum entropy RL framework becomes (Ha et al., 2020):


(3) $$\left\{ \begin{array}{l} \underset{\pi }{\max}\underset{(s_{t},a_{t})\sim \rho_{\pi }}{E}\left[\sum_{t}\gamma^{t}r(s_{t},a_{t})+\alpha ℋ(\pi_{\theta }(▪|s_{t})\right],\\ \text{s.t. }\underset{(s_{t},a_{t})\sim \rho_{\pi }}{E}\left[\sum_{t}\gamma^{t}c(s_{t},a_{t})\right]\leq C, \end{array}\right.$$

where $\underset{\left(s_{t},a_{t}\right)\sim \rho_{\pi }}{E}\left[-\log\left(\pi_{\theta }\left(a_{t}|s_{t}\right)\right)\right]\geq H_{0},\;\forall t$ so that the policy entropy satisfies the entropy constraint $H_{0}$.

A constrained optimization problem can be solved by the Lagrange multiplier method (Bertsekas, 1982). By introducing the Lagrange multiplier β, Equation (3) becomes an unconstrained optimization problem:


(4) $$\begin{array}{l} \begin{array}{l} \underset{\beta }{\min}\underset{\pi }{\max}\;L\left(\pi ,\beta \right)≐f\left(\pi \right)-\beta g\left(\pi \right)\\ \text{where }f\left(\pi \right)=\underset{\left(s_{t},a_{t}\right)\sim \rho_{\pi }}{E}[\underset{t}{\sum }\gamma^{t}r\left(s_{t},a_{t}\right)+\alpha H\left(\pi_{\theta }\left(▪|s_{t}\right)\right]\\ \text{and }g\left(\pi \right)=\underset{\left(s_{t},a_{t}\right)\sim \rho_{\pi }}{E}\left[\underset{t}{\sum }\gamma^{t}c\left(s_{t},a_{t}\right)\right]-C. \end{array} \end{array}$$

### 2.4. Transfer learning

**Figure 1:**
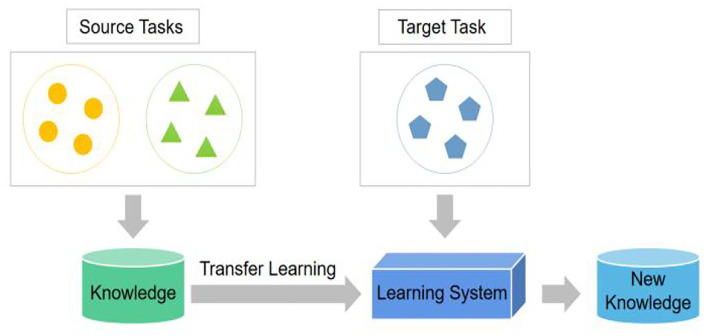
Schematic of TL.

Transfer learning (TL) is a machine learning method that improves the training efficiency for a new task by transferring knowledge about source tasks to a new task as shown in Figure 1.

Humans learn new knowledge based on the knowledge and experience accumulated by our ancestors. There are three standard TL methods: parameter-based, feature-based, and instance-based methods (Cook et al., 2013; Zhong et al., 2018; Song et al., 2020). This study applies a parameter-based TL method. When learning a target task, knowledge about source tasks can be saved as network parameters. This approach is efficient and easy to implement.

## 3. Problem formulation

This section describes the air-to-surface mission for the multi-UAV system. A kinematic model is constructed for the UAVs. Then, the decision-making process of each UAV is formulated as a CMDP.

### 3.1. Mission description

**Figure 2:**
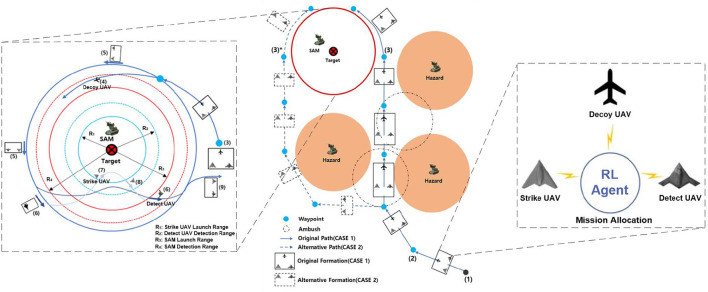
Multi-UAV cooperative decision-making scenario for the strike mission. The formation can choose the original path (case 1) or an alternative path (case 2) when flying to the SAM airspace.

The goal of the air-to-surface mission is to autonomously and cooperatively fire a missile at the opponent's surface-to-air missile (SAM) and the target (Kim et al., 2019). Suppose that the opponent has one SAM, one target, and three radar sites. The positions are known in advance through satellite imagery. The SAM is responsible for protecting important targets, such as an airport. The radar sites can detect incoming UAVs and are considered to be hazards. Our multi-UAV formation consists of three heterogeneous UAVs: a decoy UAV, a detection UAV, and a strike UAV. The decoy UAV carries decoy payloads and is tasked to lure the SAM system to turn its radar on. The detect UAV is responsible for locating the SAM and identifying the target accurately. The strike UAV will lock onto and hit the target. The three UAVs must avoid detection by maneuvering around the hazards and finally hit the target to complete the mission. Each UAV is modeled as an RL agent. Figure 2 is a schematic of this mission.

As shown in the left side of Figure 2, the detecting and weapon launch ranges of small UAVs (blue-dashed and blue solid circles) are shorter than those of the SAM (red-dashed and red solid circles). Therefore, once the multi-UAV formation reaches the SAM and target airspace, it must implement fine cooperative decisions to complete the strike mission. First, to induce the SAM radar to turn on, the decoy UAV must continually fly between the SAM detection radius *R*_4_ and the SAM launch radius *R*_3_. Second, when the SAM radar turns on, the detect UAV must quickly enter within its detection radius *R*_2_ so that it can detect and identify the target. Third, the strike UAV immediately enters within its weapon launch radius *R*_1_ and hits the target. Figure 2 is the mission scenario described by Kim et al. (2019), which inspired our study.

In conclusion, the decoy UAV needs to avoid the hazards, reach the target area, and induce the SAM radar to turn on. This is task 1. The detect UAV needs to avoid the hazards, enter within its detection radius, and detect the target. This is task 2. The strike UAV needs to avoid the hazards, enter within its launch radius, and hit the target. This is task 3. If the UAVs complete tasks 1, 2, and 3, the mission is a success. The tasks are summarized in Table 1. *D* is a distance function. *D*_1_, *D*_2_, and *D*_3_ indicate the distances between the SAM and each of the three UAVs, respectively.

**Table 1:** Task allocation.

**UAV**	**Task**	**Description**
Decoy UAV	1	*D*(DetectUAV, Hazard) > *R*_4_, *D*_1_ = *D*(DecoyUAV, SAM), *R*_3_ < *D*_1_ ≤ *R*_4_
Detect UAV	2	*D*(DetectUAV, Hazard) > *R*_4_, *D*_2_ = *D*(DetectUAV, SAM), *R*_3_ < *D*_2_ ≤ *R*_2_
Strike UAV	3	*D*(StrikeUAV, Hazard) > *R*_4_, *D*_3_ = *D*(StrikeUAV, SAM), *D*_3_ < *R*_1_

### 3.2. UAV kinematic model

A UAV is modeled using a kinematic equation with three degrees of freedom: heading, pitch, and roll. However, a UAV is not required to operate at high mobility in an actual mission due to weapon and sensor limitations. For simplicity, we developed a simple UAV kinematic model for the three UAVs as follows:


(5) $$\begin{array}{l} \left[\begin{array}{l} ẋ\left(t\right)\\ ẏ\left(t\right)\\ \overset{•}{\varphi }\left(t\right)\\ \overset{•}{v}\left(t\right) \end{array}\right]=\left[\begin{array}{l} v\left(t\right)\cos\varphi \left(t\right)\\ v\left(t\right)\sin\varphi \left(t\right)\\ \omega \left(t\right)\\ u\left(t\right) \end{array}\right], \end{array}$$

where *x* and *y* are the coordinates of the UAV in two dimensions, φ and *v* are the heading and speed of the UAV, and ω and *u* are the angular velocity and acceleration of the UAV, respectively. To ensure that each UAV has a stable heading and velocity, we controlled the angular velocity and acceleration.

In addition, the position, velocity, and heading constraints were modeled as the lower and upper bounds:


(6) $$\begin{array}{l} \{\begin{array}{l} 0\leq x\leq x_{\max},\\ 0\leq y\leq y_{\max},\\ v_{\min}\leq v\leq v_{\max},\\ \varphi_{\min}\leq \varphi \leq \varphi_{\max}. \end{array} \end{array}$$

### 3.3. CMDP modeling

**Figure 3:**
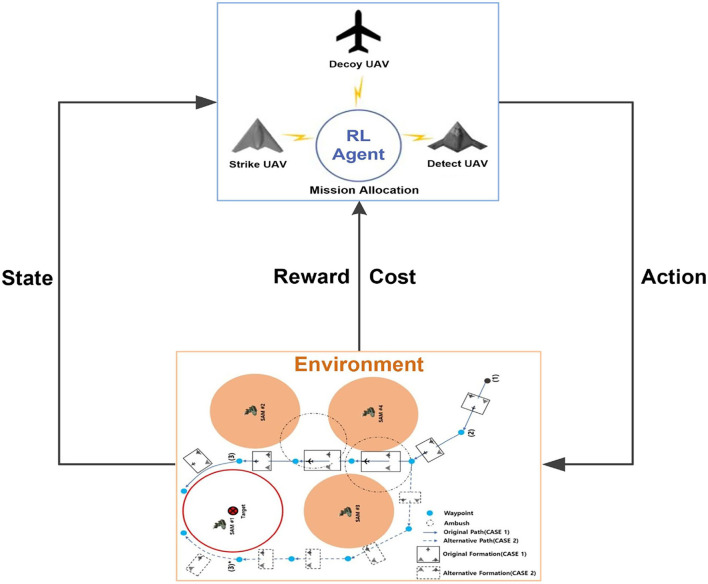
Schematic of safe RL.

Multi-UAV cooperative decision-making is a sequential decision problem and an optimization problem with safety constraints (avoiding flying into hazardous airspace). Therefore, it is mathematically formalized as a CMDP. Figure 3 shows the interactions between the agent and the environment in CMDP.

In this study, each UAV is an agent interacting with the environment, which is modeled as a CMDP. The state space, action space, reward function, and cost function are designed as follows.

#### 3.3.1. State space

In the CMDP, the state space should contain the critical information needed for the agent to make decisions. It evaluates the agent's current situation and then guides it to complete the task. Therefore, the state space is designed as follows:


(7) $$\begin{array}{l} s_{t}=\left[x_{t},y_{t},v_{t},\varphi_{t},l_{t}\right], \end{array}$$

where *x*_*t*_ and *y*_*t*_ are the coordinates of a UAV at time *t*, *v*_*t*_ and φ_*t*_ are the speed and heading, and *l*_*t*_ is the distance between the UAV and the target. The target position is detected by satellites and the relative distance can be obtained with the radar sensors on the UAV.

#### 3.3.2. Action space

The agent selects an action and transitions to the next state. The state transition function is the UAV kinematics model as shown in Equation (5). Therefore, the action space is designed as follows:


(8) $$\begin{array}{l} a_{t}=\left[\omega_{t},u_{t}\right]. \end{array}$$

By directly controlling ω and *u*, the UAV can maintain a reasonable speed and a stable heading to accomplish the mission better.

#### 3.3.3. Reward function

The reward function is a feedback signal from the environment. It is used to evaluate the actions of an agent. An agent receives a reward signal only when it completes a task, but a sparse reward is not conducive to learning the policy for an agent. Therefore, a dense reward function is designed according to expert knowledge.

The reward function in this study is divided into three parts: a positive reward $r_{1}^{\text{done}}$ for the success of the task, a dense reward $r_{2}^{\text{approach}}$ for inducing the agent to approach the target, and a negative reward $r_{3}^{\text{step}}$ for the time consumed by the agent in completing the task, as shown in Table 2.

**Table 2:** The settings of the reward function.

**Description**	**Reward function**
Success reward	$r_{1}^{\text{done}}=10$
Distance reward	$r_{2}^{\text{approach}}=d_{t-1}-d_{t}$
Step penalty	$r_{3}^{\text{step}}\text{=}-0.1$

Here, *d*_*t*−1_ and *d*_*t*_ are the distances between the UAV and the SAM at the previous and current time steps. When *d*_*t* − 1_ > *d*_*t*_, the UAV is close to the SAM, and therefore, $r_{2}^{\text{approach}}>0$; when *d*_*t*−1_ < *d*_*t*_, the UAV is far from the SAM, and therefore, $r_{2}^{\text{approach}}<0$.

In addition, the three UAVs receive $r_{1}^{\text{done}}$ under different conditions. The decoy UAV receives $r_{1}^{\text{done}}$ when it completes task 1 (it enters the area between the detection radius and launch radius *R*_3_ of the SAM). The detect UAV receives $r_{1}^{\text{done}}$ when both the decoy UAV has completed task 1 and it completes task 2 (the distance between the detect UAV and the target is less than its detection range *R*_2_). The strike UAV obtains $r_{1}^{\text{done}}$ when both it completes task 3 and the detect UAV has completed task 2. Therefore, $r_{1}^{\text{done}}$ is given as follows:


(9) $$\begin{array}{l} r_{1}^{\text{done}}=\{\begin{array}{l} 10\text{to Decoy UAV},\text{if}R_{3}<D_{1}\leq R_{4},\\ 10\text{to Detect UAV},\text{if}R_{3}<D_{1}\leq R_{4}\\ \text{and}R_{1}<D_{2}\leq R_{2},\\ 10\text{to Strike UAV},\text{if}R_{3}<D_{1}\leq R_{4}\\ \text{and}R_{1}<D_{2}\leq R_{2}\text{and}D_{3}<R_{1}. \end{array} \end{array}$$

The overall reward function is the sum of the three subrewards:


(10) $$\begin{array}{l} r=k_{1}r_{1}^{\text{done}}+k_{2}r_{2}^{\text{approach}}+k_{3}r_{3}^{\text{step}}, \end{array}$$

where *k*_1_, *k*_2_, and *k*_3_ are the weights.

#### 3.3.4. Cost function

The cost function improves the safety of the agent. For each step during training, if the agent takes an unsafe action or moves within range of the hazards, it receives a cost *c*. The cumulative costs of the agent during training cannot exceed the cost threshold *C*. Therefore, the agent must make relatively few unsafe actions to satisfy the safety constraint when exploring. The cost function is defined as follows:


(11) $$\begin{array}{l} c=\{\begin{array}{l} 1,\text{ if the UAV stays within range of the hazards,}\\ 0,\text{ else.} \end{array} \end{array}$$

## 4. Method

For the multi-UAV cooperative decision-making problem, this study used a SAC (Haarnoja et al., 2018a), which is an efficient maximum entropy RL algorithm. To improve the safety of each UAV during exploration, an improved safe RL algorithm was used to solve the CMDP model established in the “CMDP modeling” section. However, it is challenging to train multiple UAVs simultaneously. Moreover, learning by the multi-agent system is inefficient. Thus, considering the similarity and increasing difficulty among the three tasks, a parameter-based TL method was used to train the detect UAV and strike UAV models based on the converged decoy UAV and detect UAV models, respectively. Finally, the TSSAC algorithm is proposed to improve training efficiency.

### 4.1. SAC-Lagrangian

SAC is a model-free, off-policy, actor-critic algorithm following the maximum entropy RL framework. It is sample-efficient and robust. Therefore, we used SAC to train agents in this study.

SAC-Lagrangian, namely SAC combined with the Lagrange multiplier method, is an efficient safe RL algorithm. The loss functions used for Equation (4) are given below. Their derivation is described in detail in the literature (Haarnoja et al., 2018b; Ha et al., 2020).

The policy loss is:


(12) $$\begin{array}{l} J_{\pi }(\theta )=\underset{(s_{t},a_{t})\sim \rho_{\pi }}{E}[\alpha \log\pi_{\theta }(a_{t}|s_{t})-Q_{w}^{r}(s_{t},a_{t})\\ \;+\beta Q_{w}^{c}(s_{t},a_{t})], \end{array}$$

where *w* represents the parameters of the critic and β is the safety coefficient or Lagrange multiplier. We used $Q_{w}^{r}\left(s_{t},a_{t}\right)$ to represent the reward critic and $Q_{w}^{c}\left(s_{t},a_{t}\right)$ for the safety critic.

The soft *Q* function is trained to minimize the soft Bellman residual. The critic loss can be expressed by the following equation:


(13) $$\begin{array}{l} J_{Q}(w)=\underset{(s_{t},a_{t})\sim \rho_{\pi }}{E}[\frac{1}{2}(Q_{w}(s_{t},a_{t})-(r(s_{t},a_{t})\\ \;+\gamma \underset{s_{t+1}\sim \rho_{\pi }(s)}{E}[V_{\bar{\theta }}(s_{t+1})]){)}^{2}], \end{array}$$

where $V_{\bar{\theta }}$ is the target value function.

The entropy constraint loss function is:


(14) $$\begin{array}{l} J\left(\alpha \right)=\underset{\left(s_{t},a_{t}\right)\sim \rho_{\pi }}{E}\left[-\alpha \log\pi_{\theta }\left(a_{t}|s_{t}\right)-\alpha H_{0}\right], \end{array}$$

where $H_{0}$ is the minimum expected entropy.

We learn β by minimizing the safety loss function as Equation (15). So β will be decreased if $Q_{w}^{c}\left(s_{t},a_{t}\right)\leq C$, else β will be increased to guarantee more safety.


(15) $$\begin{array}{l} J\left(\beta \right)=\underset{\left(s_{t},a_{t}\right)\sim \rho_{\pi }}{E}\left[\beta \left(C-Q_{w}^{c}\left(s_{t},a_{t}\right)\right)\right]. \end{array}$$

The policy is learned by solving the corresponding gradient of the loss function and updating the network parameters using the gradient descent algorithm.

### 4.2. Modified Lagrange multiplier method

The Lagrange multiplier method transforms the constrained optimization problem into a dual problem through a linear combination of the objective function and constrained condition, as shown in Equation (4).

**Figure 4:**
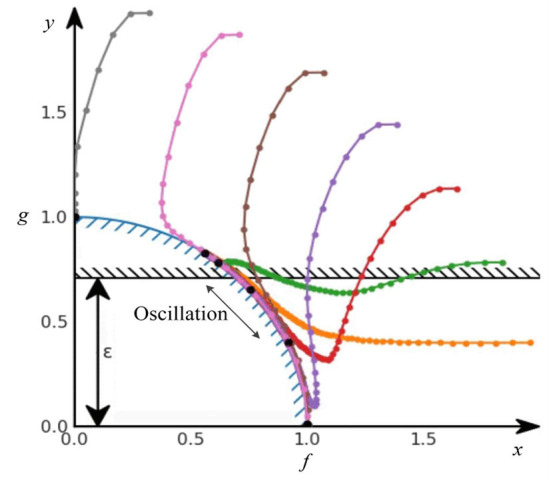
Oscillations on the concave Pareto front.

However, it never converged into a solution in practice. As long as the constraint was violated, β kept increasing. However, when we suddenly satisfied the constraint again, β remained large. It took several steps before the gradient descent pushed β back to zero. As long as β was positive, the solution was pushed further away from the constraint. Eventually, β became zero, the constraint was ignored, and the optimization process continued. However, when the solution accidentally hit the constraint again, the whole cycle repeated. This optimization method oscillated on the Pareto front, especially on the concave Pareto front. Figure 4 shows the standard Lagrange multiplier method oscillating on the Pareto front.

Therefore, we introduced a modified differential method of multipliers proposed by Platt and Barr (1987), which is the modified Lagrange multiplier method. Intuitively, the Lagrange multiplier β can be viewed as the potential energy of an oscillating system. By damping this energy, we can prevent the system from oscillating eternally and make it a tunable algorithm for the stochastic gradient descent and SAC-Lagrangian. Therefore, Equation 12 becomes:


(16) $$\begin{array}{l} J_{\pi }\left(\theta \right)=\underset{\left(s_{t},a_{t}\right)\sim \rho_{\pi }}{E}[\alpha \log\pi_{\theta }\left(a_{t}|s_{t}\right)-Q_{w}^{r}\left(s_{t},a_{t}\right)\\ \;+\left(\beta -\xi \right)Q_{w}^{c}\left(s_{t},a_{t}\right)], \end{array}$$

(17) $$\begin{array}{l} \xi =k_{d}\times \left(C-Q_{w}^{c}\left(s_{t},a_{t}\right)\right), \end{array}$$

where ξ is a damping factor and *k*_*d*_ is a damping scale coefficient.

We used this modified Lagrange multiplier method to tune the balance between the losses using a stochastic gradient descent, regardless of the shape of the invisible Pareto front. This approach requires significantly less effort for tuning the hyperparameters. The optimization procedures do not need to be iterated as much, and they will be more robust to the initial parameter values too.

### 4.3. TSSAC

We used a parameter-based TL method to improve the training efficiency of SAC-Lagrangian, which is called TSSAC. In practice, the model trained on the source task was saved and transferred as the initialization of the target task model to accelerate learning and improve the training efficiency. The target task model continues to be trained to gain new knowledge.

**Figure 5:**
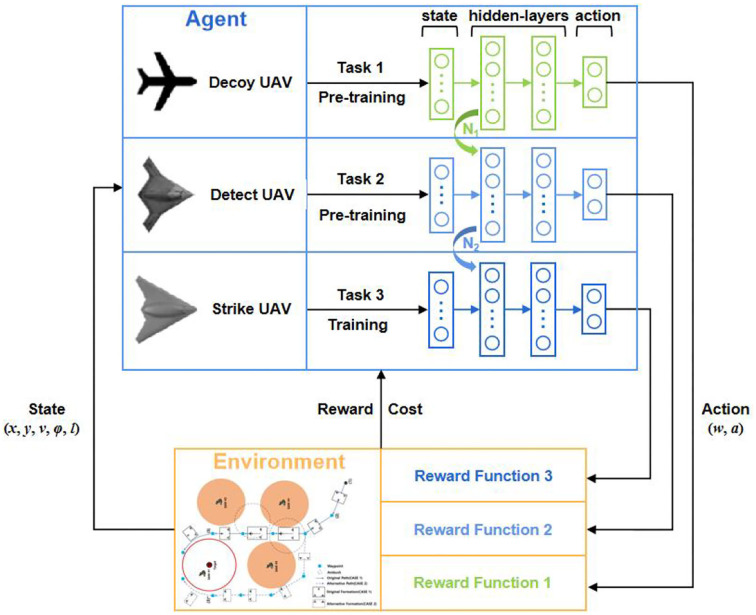
Overview of our proposed framework.

Therefore, the training flow of this study is as follows. First, the decoy UAV model was trained on task 1, and the weights and bias parameters of the current networks were saved as *N*_1_. Second, we loaded the trained *N*_1_ as the initialization of the detect UAV network, and the detect UAV model was trained on task 2. We loaded the decoy UAV model as the judgment condition for the completion of task 2. The weights and bias parameters of the networks were saved as *N*_2_. Finally, we loaded converged *N*_2_ as the initialization of the strike UAV network, and the strike UAV model was trained on task 3. It should be noted that we loaded the decoy UAV and detect UAV models as the judgment condition for the completion of task 3. The research framework for multi-UAV cooperative decision-making based on TSSAC was depicted in Figure 5. Algorithm 1 shows the details of the TSSAC algorithm.

**Algorithm 4.1:** Transfer-safe soft actor-critic algorithm (TSSAC).

1. Decompose the cooperative task as Table 1 into three related subtasks: 1, 2, and 3; load task 1;
2. Initialize the network parameters of the replay buffer: *M, Q*, π, α, β;
3. Initialize the target network weights: *Q*′;
4. **For** each epoch **do**
5. Initialize state *s*_0_ for the agent;
6. **For** each environment step **do**
7. Sample the action from the policy π;
8. Execute action *a*_*t*_ and get the next state *s*_*t*+1_ and reward *r*_*t*_ from the environment;
9. Store the tuple *s*_*t*_, *a*_*t*_, *r*_*t*_, *s*_*t*+1_, *d*_*t*_ to *M*;
10. **End for**
11. **For** each gradient step **do**
12. Randomly sample a batch of memories from *M*;
13. Update the policy (Equation 12), the *Q* network (Equation 13), the temperature parameter (Equation 14), the cost coefficient (Equation 15), and the target network weights (soft update);
14. **End for**
15. **End for**
16. Save the network parameters as *N*_1_ and load task 2;
17. Transfer: *N*_1_ → *N*_2_, fine-tune the hyperparameters, **do** second pretraining in 2–15;
18. Save network parameters as *N*_2_ and load task 3;
19. Transfer *N*_2_ → *N*_3_, fine-tune the hyperparameters, **do** training in 2–15.

Thus, we obtained a multi-UAV cooperative decision-making model. For online testing, the three trained UAV models were loaded simultaneously to verify the performance of the overall model.

## 5. Experiments and discussion

### 5.1. Experimental settings

**Figure 6:**
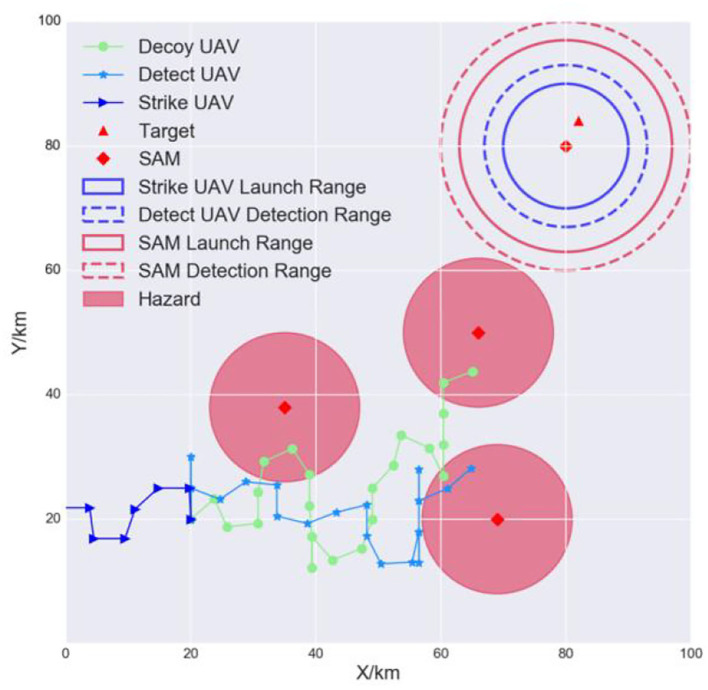
Simulation environment.

A light multi-UAV cooperative decision-making environment was constructed in this study. The scenario is a square of area 100 km × 100 km. The maximum range of the decoy UAV, detect UAV, and strike UAV is 20 km, 13 km, and 10 km, respectively. The SAM launch, detection, and other hazards ranges are 17 km, 20 km, and 12 km, respectively, as shown in Figure 6.

The size of the scenario was normalized to the range (0, 1) during training, which facilitates neural network training and prevents the gradient from vanishing. The angular velocity and acceleration ranges were both normalized to [−1, 1], squashed by the activation function tanh. The simulation step size was 0.1 s. The hyperparameter settings are listed in Table 3. The Adam optimizer was employed for gradient descent optimization (Kingma and Ba, 2014).

**Table 3:** Simulation parameters.

**Hyperparameter**	**Value**	**Symbol**
State dimension	5	
Action dimension	2	
Number of layers for actor	3	
Number of layers for critic	3	
Number of nodes for actor	(256, 256, 2)	
Number of nodes for critic	(256, 256, 1)	
Active function	ReLU	
Learning rate	0.001	η
Reward decay factor	0.99	γ
Max step per epoch	4,000	
Epochs	50	
Replay size	1,000,000	*M*
Polyak	0.995	τ
Minibatch size	256	
Entropy constraint	−1.0	$H_{0}$
Cost threshold	3	*C*
Cost per step for hazards	1	γ
Initial entropy coefficient	0	α
Initial cost coefficient	0	β
Damping scale factor	10	*k*_*d*_
Number of seeds	3	
Reward function weights	(1, 1, 1)	(*k*_1_, *k*_2_, *k*_3_)
Strike UAV launch range	10	*R*_1_
Detect UAV detection range	13	*R*_2_
SAM launch range	17	*R*_3_
SAM detection range	20	*R*_4_
Simulation step (second)	0.1	

### 5.2. Results and discussion

We ran experiments using the TSSAC method in the simulation environment. First, we trained the decoy UAV, detect UAV, and strike UAV. We recorded the rewards and costs during training and testing and compared them with the results from proximal policy optimization (PPO) (Schulman et al., 2017), soft actor-critic (SAC), constrained policy optimization (CPO) (Achiam et al., 2017), and SAC-Lagrangian benchmark algorithms. Both PPO and SAC are conventional RL algorithms, whereas CPO and SAC-Lagrangian are safe RL algorithms. Finally, we tested online the effectiveness, generalization, and trajectories of the agents for the different algorithms.

#### 5.2.1. Training results

**Figure 7:**
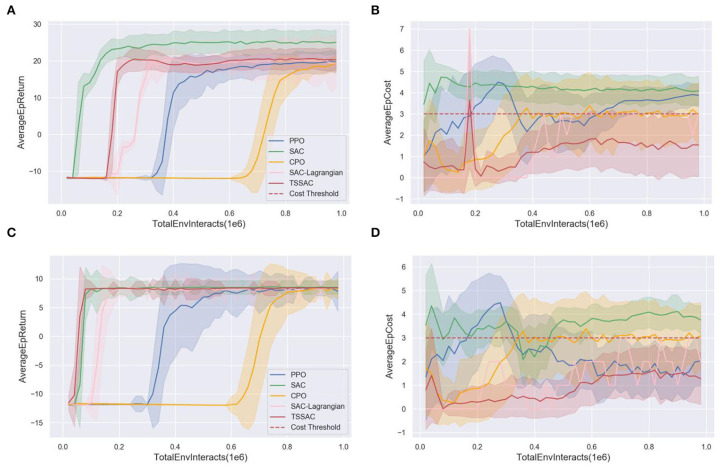
Comparison of the five algorithms during training. **(A)** The task performance curve of the detect UAV. **(B)** The safety curve of the detect UAV. **(C)** The task performance curve of the strike UAV. **(D)** The safety curve of the strike UAV.

After training 50 epochs, the task performance (average epoch return) and safety curves (average epoch costs) during the training of the five algorithms were obtained. The task performance and safety curves of the detect UAV and the strike UAV are shown in Figure 7.

Figures 7A, C show that the three SAC-based algorithms outperformed the PPO and CPO algorithms in terms of convergence speed and final performance. The maximum entropy framework and stochastic actor of SAC enhance the exploration capability of the agent and the off-policy improves the sample efficiency. Due to the combination of safety constraints, SAC-Lagrangian has a lower final task performance and learning efficiency than SAC. The TSSAC algorithm uses TL, so that learning is more efficient and convergence is faster while ensuring that the final performance is the same as that of SAC-Lagrangian.

The red dashed lines in Figures 7B, D are the cost threshold. The SAC and PPO algorithms violate the cost threshold because they maximize only the rewards and do not consider the safety constraint. Therefore, the cumulative costs violate the cost threshold. In contrast, the CPO, SAC-Lagrangian, and TSSAC algorithms, which consider the safety constraint, all satisfy the cost threshold. The CPO and SAC-Lagrangian algorithms reach the cost threshold. TSSAC performed better because it is based on the pretrained SAC-Lagrangian model as the agent's policy is more conservative and safer.

**Figure 8:**
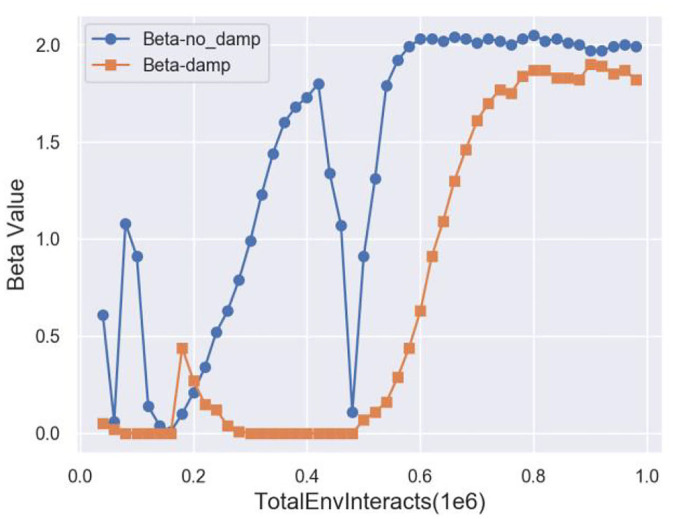
Cost coefficient curve.

Figure 8 plots the cost coefficient β. After adding damping, β is more stable. Therefore, the performance curve of TSSAC converges more easily.

#### 5.2.2. Experiment I: Effectiveness

**Figure 9:**
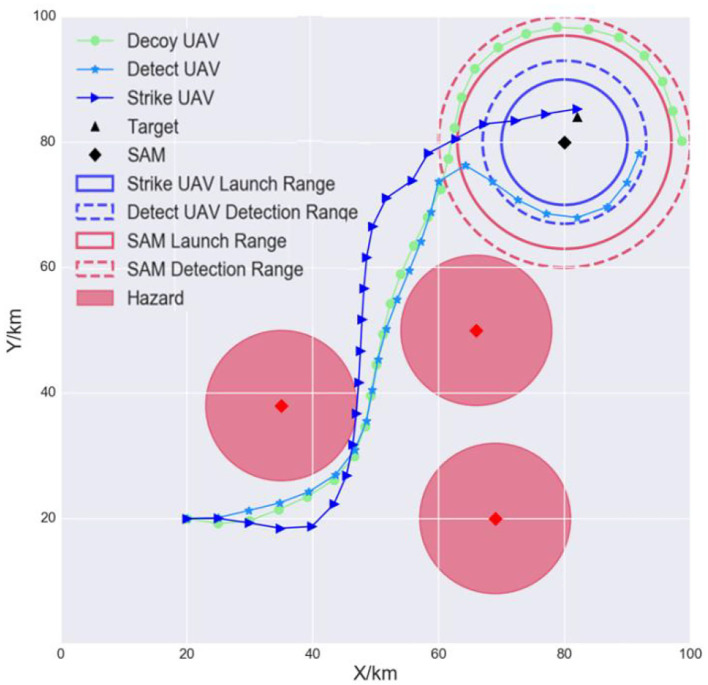
Effectiveness during testing.

We loaded the trained model and tested it online to verify the effectiveness of TSSAC. Figure 9 shows that the decoy UAV can avoid the hazards and then fly stably between the detection and launch ranges of the SAM to induce the SAM to turn its radar on. The detect UAV can also avoid hazards. At the same time, it locates the SAM and the target when they are within its detection range. The strike UAV adjusts its heading and waits for the detect UAV to locate and identify the target and then quickly hit it. The target turns black after being hit. Therefore, the three trained UAV models can engage in online cooperative decision-making to accomplish their tasks well.

**Figure 10:**
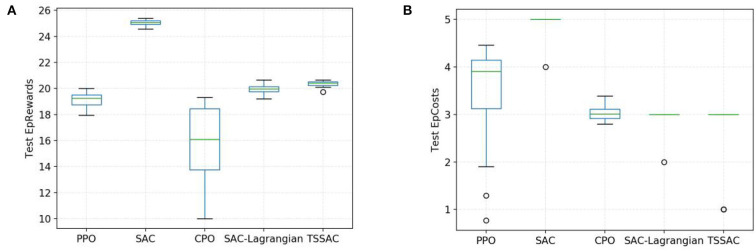
Comparison of five algorithms during testing. **(A)** Task performance/reward test. **(B)** Safety test.

We also tested the distribution of rewards and costs, as shown in the box plots in Figure 10. Figure 10A indicates that the task performance/reward during testing of the five algorithms was consistent with that during training as shown in Figure 7A. The CPO algorithm results in a large variance in reward distribution and poor task performance due to its learning inefficiency. The SAC-based algorithm has a smaller variance for reward distribution and more stable and excellent performance.

The TSSAC algorithm achieves better final performance and stability than the SAC-Lagrangian algorithm due to TL.

Figure 10B shows that the costs of the PPO, SAC, and CPO algorithms exceed the cost threshold during testing, whereas the SAC-Lagrangian and TSSAC algorithms are both within the cost threshold. Therefore, the TSSAC algorithm is safer than these algorithms and can guarantee safety during testing.

We randomly initialized the scenario layout and ran the test 50 times to compare the success rates of the five algorithms. The success rate is calculated as the number of times all three tasks were successful as a percentage of the total number of simulation runs. The results are shown in Table 4.

**Table 4:** Comparison of test performance.

**Algorithm**	**Success rate**	**Average cost**
PPO	92%	3.8
SAC	98%	5.0
CPO	76%	3.0
SAC-Lagrangian	96%	3.0
TSSAC	96%	3.0

Table 4 shows that the SAC algorithm has the highest success rate but the highest average cost as it sacrifices safety. Both CPO and PPO have lower success rates due to lower returns. However, TSSAC had the same success rate of 96% as SAC-Lagrangian while also guaranteeing safety.

#### 5.2.3. Experiment II: Generalization

**Figure 11:**
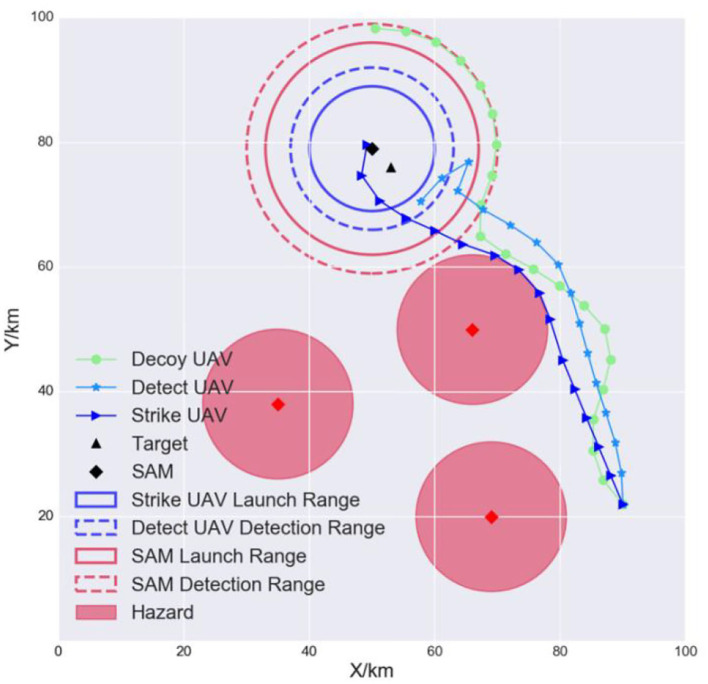
Generalization of the models.

In a real-world scenario, the UAVs need to strike dynamic and time-sensitive targets. Therefore, we tested the generalization of the models when the target and the SAM were in a different initial position. The results are shown in Figure 11. The models were still able to complete their tasks cooperatively. This is because they were trained with different initial positions. For each epoch, the layout was randomly initialized, so the agents were implicitly trained for different scenarios. Therefore, the TSSAC algorithm is robust and can adapt to uncertain scenarios.

#### 5.2.4. Experiment III: Safety

**Figure 12:**
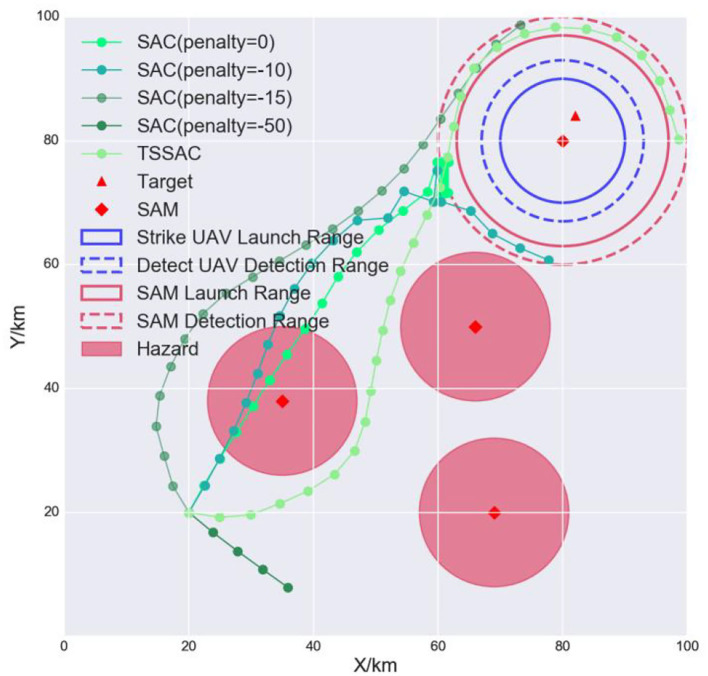
Safety analysis of the UAV trajectories.

To analyze the safety issues, we tested the safety of the conventional RL algorithm SAC and the safe RL algorithm TSSAC. SAC applies hazard penalties for unsafe actions as negative rewards, which can improve safety to a certain extent. The resulting trajectories of the agents are shown in Figure 12.

The SAC algorithm achieves some degree of safety by setting a penalty. When the penalty is 0, the agent ignores the first hazard and directly crosses the hazard region. When the penalty is −10, the agent slightly avoids the hazard region to increase the task reward. When the penalty is −15, the agent completely avoids the hazard but maneuvers too far from it, which reduces the task reward, and so the agent behaves conservatively. When the penalty is −50, the agent completely avoids the hazard to maximize the reward but does not complete the task. Therefore, it is hard to balance effectiveness and safety by setting penalties. In contrast, TSSAC uses the hazard as a safety constraint. The agent was able to ensure that the cost threshold was not violated during training and can realize a trade-off between reward and safety. It completes the task while guaranteeing safety.

## 6. Conclusion

In this study, we present an end-to-end TSSAC method for multi-UAV cooperative decision-making. The decision-making process for each UAV is modeled as a CMDP. An improved SAC-Lagrangian algorithm was used to solve it, which improves the safety of the UAVs. A multi-UAV cooperative decision-making framework combining TL is proposed to improve the training efficiency of a multi-UAV system. The experimental results demonstrate the effectiveness, generalization, and safety of the proposed TSSAC approach during training and testing. Therefore, TSSAC enables a multi-UAV system to adapt to dynamic changing scenarios and achieve cooperative decisions to accomplish complicated tasks.

In the future, we will apply this method to a high-fidelity digital simulation environment to enhance the adversarial game for both sides. Furthermore, we will study and propose new efficient cooperative decision-making methods for large-scale multi-agent UAV swarms.

## Data availability statement

The raw data supporting the conclusions of this article will be made available by the authors, without undue reservation.

## Author contributions

Conceptualization: LY and JZ. Methodology: LY and YZ. Formal analysis: JZ. Writing: LY. Supervision: RY. Project administration: YZ. All authors have read and agreed to the published version of the manuscript.
